# Awareness and Acceptability of Prenatal Diagnosis of Sickle Cell Disease among Mothers of Affected Children in a Northern Nigerian Teaching Hospital

**DOI:** 10.12688/openreseurope.18700.2

**Published:** 2025-12-09

**Authors:** Aliyu RM, Sada SI, Adebiyi NM, Randawa AJ, Abdulkadir I, Ahmad HR

**Affiliations:** 1 Department of Obstetrics and Gynaecology, Ahmadu Bello University Faculty of Medicine, Zaria, Kaduna, Nigeria; 2 Department of Obstetrics and Gynaecology, Ahmadu Bello University Teaching Hospital, Zaria, Kaduna, Nigeria; 3 Department of Paediatrics, Ahmadu Bello University Teaching Hospital, Zaria, Kaduna, Nigeria; 4 Department of Paediatrics, Ahmadu Bello University, Zaria, Kaduna, Nigeria

**Keywords:** awareness, acceptability, prenatal diagnosis, mothers, sickle cell disease.

## Abstract

**Background:**

Nigeria has the highest global prevalence of sickle cell disease (SCD), necessitating effective preventive measures to control the disease. Prenatal diagnosis (PND) is a key early intervention for SCD, yet there is a shortage of studies in Northern Nigeria focusing on mothers of children with SCD. These mothers not only carry the burden of the disease but also face the risk of having more affected children, making them vital stakeholders in managing and controlling SCD. This study assessed the awareness and acceptability of prenatal diagnosis PND for SCD among mothers of children with the disease.

**Methods:**

A cross-sectional study was conducted involving 297 mothers of children with SCD attending the Paediatric Haematology clinic at Ahmadu Bello University Teaching Hospital, Zaria, Nigeria. Data were collected via an interviewer-administered questionnaire addressing the characteristics of the children and mothers, as well as their awareness, attitudes, and acceptability of PND. SPSS version 23 was used for data analysis, with a p-value < 0.05 considered statistically significant.

**Results:**

Most participants (90.9%) were of Hausa ethnicity and 97.0% were of Islamic faith. A majority (77.8%) had less than tertiary education, and 57.9% lacked personal income. Nearly 41% had more than one child affected by SCD, and about one-fifth had lost a child to SCD complications. Only 22.2% of mothers had heard of PND, mostly from healthcare workers, and just 0.3% had undergone the procedure. After receiving information about PND, 93.3% were willing to accept it, though 45% of those who declined cited religious beliefs. Factors associated with PND acceptance included the mother's education and the child’s SCD severity.

**Conclusion:**

Awareness of PND among at-risk mothers is low, yet most expressed willingness to accept it. Educational level and disease severity were associated with PND acceptability.

## Plain language summary

Nigeria has the highest number of people with sickle cell disease (SCD) in the world. To help prevent more children from being born with the disease, prenatal diagnosis (PND) allows parents to know whether their baby will have SCD before birth. However, there is little research in Northern Nigeria focusing on mothers who already have children with SCD. These mothers carry the heavy burden of caring for sick children and face the possibility of having more children with the disease. Understanding how much these mothers know about PND and whether they would be willing to use it is important for controlling the spread of SCD. In this study, 297 mothers of children with SCD attending a hospital in Zaria, Nigeria, were surveyed. The mothers answered questions about their children, personal background, and knowledge of and willingness to accept PND. The data were analyzed to identify factors that influenced awareness and acceptability of PND. The majority of the mothers were Hausa and Muslim, and most had low levels of education. Over half of the mothers had no personal income. Nearly 41% of the mothers had more than one child with SCD, and about 20% had lost a child due to the disease. Only 22.2% of the mothers had heard of PND, mostly from healthcare workers, and just 0.3% had undergone the procedure. After learning more about PND, 93.3% of the mothers said they would be willing to use it. However, religious belief was the main reason for refusal among those who declined. Mothers with higher education levels and those whose children had more severe disease manifestation were more likely to accept PND. In conclusion, awareness of PND among these mothers is low, but most are open to it once informed. Educational efforts could improve acceptance of PND in Northern Nigeria.

## Introduction

Sickle cell disease (SCD) is the most common hemoglobinopathy in sub-Saharan Africa (SSA) with Nigeria having the highest prevalence globally ^1, 2^. The World Health Organisation (WHO) has identified SCD as a public health burden due to its high morbidity and mortality rates. In SSA, most affected children die before their fifth birthday, and survivors face recurrent episodes of anemia, blood transfusions, crises, and repeated hospital admissions. These not only cause physical ill-health but also lead to significant psychosocial trauma for affected individuals and their families, creating a substantial socio-economic burden on families, society, and the country ^3^. Therefore, preventing SCD is essential to reduce its overall burden.

Early diagnosis of SCD is a key strategy in controlling the disease, as it allows for the timely implementation of appropriate and acceptable measures. Screening for SCD can occur at various stages of life, with prenatal diagnosis (PND) being one of the earliest available options. PND is performed during early pregnancy for couples at risk of having children with SCD. This diagnosis can be conducted using invasive tests, such as chorionic villus sampling or amniocentesis, and more recently, a non-invasive technique has been developed. The non-invasive prenatal test screens for fetal SCD using cell-free fetal DNA from maternal plasma, eliminating the risk of miscarriage associated with invasive tests ^3, 4^. This method also allows for earlier screening than conventional invasive tests.

There is a scarcity of centers offering PND in Nigeria, and its acceptability is often influenced by religious beliefs ^5^. The benefits of PND include early detection of abnormalities in the fetus, allowing a couple time to prepare psychologically, socially, and financially for the birth of an affected child if they choose to continue the pregnancy. It also provides the option to terminate the pregnancy if the birth of an affected child is not desired, in situations where pregnancy termination is acceptable and permissible. Additionally, PND can relieve anxiety early in pregnancy if the disease is excluded.

Although some studies have been conducted on the knowledge and acceptability of PND of SCD in the general population, there is a lack of research in Northern Nigeria focusing on mothers of children with SCD. These mothers are already carrying the burden of the disease, are at risk of having another affected child, and are important stakeholders in SCD control. This study aims to assess the awareness and acceptability of PND of SCD among mothers of children with SCD.

## Methods

### Study setting

The study was conducted in the Paediatric Haematology clinic of Ahmadu Bello University Teaching Hospital, Zaria, which operates weekly on Wednesdays. The clinic sees an average of 50 children with SCD each week.

### Study design

This was a cross-sectional descriptive study.

### Study population

The study population included mothers of children with SCD who were attending the clinic.

### Inclusion criteria

All consenting mothers of at least one child living with sickle cell disease were included in the study.

### Exclusion criteria

Exclusion criteria were menopausal mothers, mothers who had undergone bilateral tubal ligation, and mothers whose current spouse had an Hb genotype of AA.

### Sample size determination

Using a prevalence of awareness of 0.25% among mothers of affected children as reported by Olatunya ^6^, where n = sample size; Z = standard normal variate (1.96 at P<0.05); p = percentage of awareness of prenatal diagnosis (0.25); q = 1-p (0.75), d = absolute precision (0.05), a minimum sample size of 288 was obtained. However, 297 women were recruited for the study.

### Sampling technique

A non-probability sampling method was used to sequentially recruit eligible mothers of affected children from the clinic as they presented to access care until the desired sample size was achieved. Recruitment took place from March to August, 2023.

### Study protocol

Every eligible participant completed a structured interviewer-administered questionnaire, after obtaining informed verbal consent, to gather data on socio-demographic characteristics, awareness, attitude, and acceptability of prenatal diagnosis for SCD. None of the eligible participants declined participation. The data collected was pseudo-anonymized. Recruitment took place from March to August 2023.

### Data analysis

Data were analysed using the Statistical Package for Social Sciences (IBM SPSS statistics) version 23 ^6^. The level of significance was set at <0.05. Frequencies and percentages were used to describe categorical data, and Chi-square/Fisher's exact test was used to test for associations between variables.

### Ethics and consent

Ethical approval (ABUTHZ/HREC/H32/2022) was obtained from the Health Research Ethics Committee of ABUTH, Zaria, on 14 September 2022. Participants were provided with adequate information, and informed verbal consent was obtained from all the study participants before the questionnaires were administered. Verbal consent was used because it has been established in a national document that Kaduna state (where the study was conducted) has a low literacy level, with only 18% of the female population reported to have attained secondary or higher education (National Health Demographic Survey, 2018). The study also posed a no-minimal risk to the participants and the information collected was de-identified. The scope of the study and process involved was discussed with each participant. The participant was allowed the opportunity to ask questions and her concerns addressed if any before giving consent. The questionnaires were de-identified, and confidentiality was strictly maintained. Collected hard copy data was securely stored, and only the principal investigator had access to the input data on a computer.

## Results

A total of 297 mothers of children with SCD were recruited. The majority of the mothers (90.9%) were of Hausa ethnicity, 97.0% were of Islamic faith, 77.8% had below tertiary education, and 57.9% had no source of personal income. About one-third of the mothers were grandmultiparous women (≥5 deliveries). Additionally, 40.7% had more than one child affected by SCD, and about one-fifth had experienced the loss of at least one child due to SCD-related complications. Further details are provided in Table 1.

**Table 1.** Socio-demographic characteristics of mothers of children with SCD.

Characteristic	Frequency (%)	Characteristic	Frequency (%)
**Tribe**		**Religion**	
Hausa	270 (90.9)	Islam	288 (97,0)
Non-Hausa	27 (9.1)	Christianity	9 (3.0)
**Educational level**		**Parity**	
Below tertiary	231 (77.8)	<5	186 (62.6)
Tertiary *	66 (22.2)	≥5	111 (37.4)
**Residence**		**No. of children with SCD**	
Rural	79 (26.6)	1	176 (59.3)
Semi-urban/urban	218 (73.4)	≥1	121 (40.7)
**Personal source of income**		**No. of sickle cell related deaths**	
Yes	125 (42.1)	0	235 (79.1)
No	172 (57.9)	≥1	62 (20.9)

*Tertiary: additional education beyond secondary level such as diplomas, degrees, postgraduate

Two-thirds of the affected children were aged five years or younger. The predominant hemoglobin genotype among the children was Hb SS (99%). Approximately half of the children experienced at least one crisis per month. In the past year, 55.2% of the children were hospitalized, and 44.4% received blood transfusions due to SCD-related complications. Detailed characteristics of the affected children attending the SCD clinic are provided in Table 2.

**Table 2.** Characteristics of the affected children attending the SCD clinic.

Characteristic	Frequency (%)
**Age (years)**	
≤5	197 (66.3)
>5	100 (33.7)
**Haemoglobin genotype**	
SS	294 (99.0)
SC	3 (1.0)
**Average crisis per month**	
0	146 (49.2)
≥1	151 (50.8)
**SCD-related blood transfusion** **in the last one year**	
0	165 (55.6)
≥1	132 (44.4)
**SCD-related hospital** **admission in the last one year**	
0	133 (44.8)
≥1	164 (55.2)

**Figure 1.**
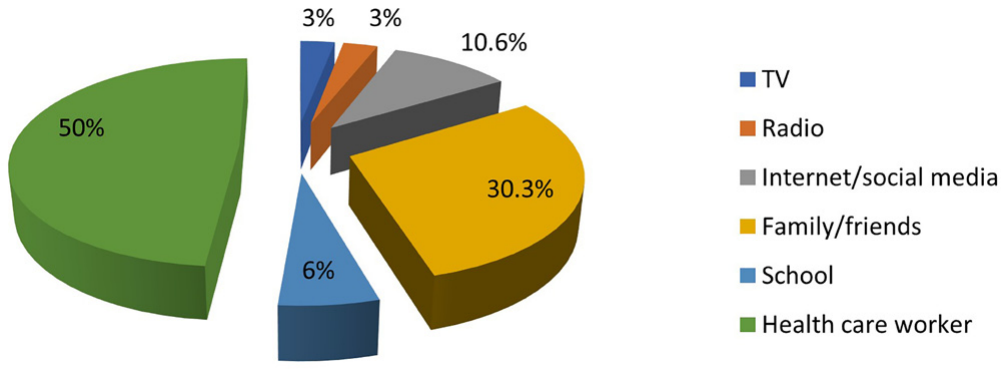
Sources of information about PND for SCD among mothers of affected children. The colours represent the different sources of information about PND.

Only 22.2% of the mothers (66 out of 297) had ever heard of PND for SCD. The most common source of information about PND was healthcare workers, while the least common source was electronic media. This is illustrated in Figure 1.

**Figure 2.**
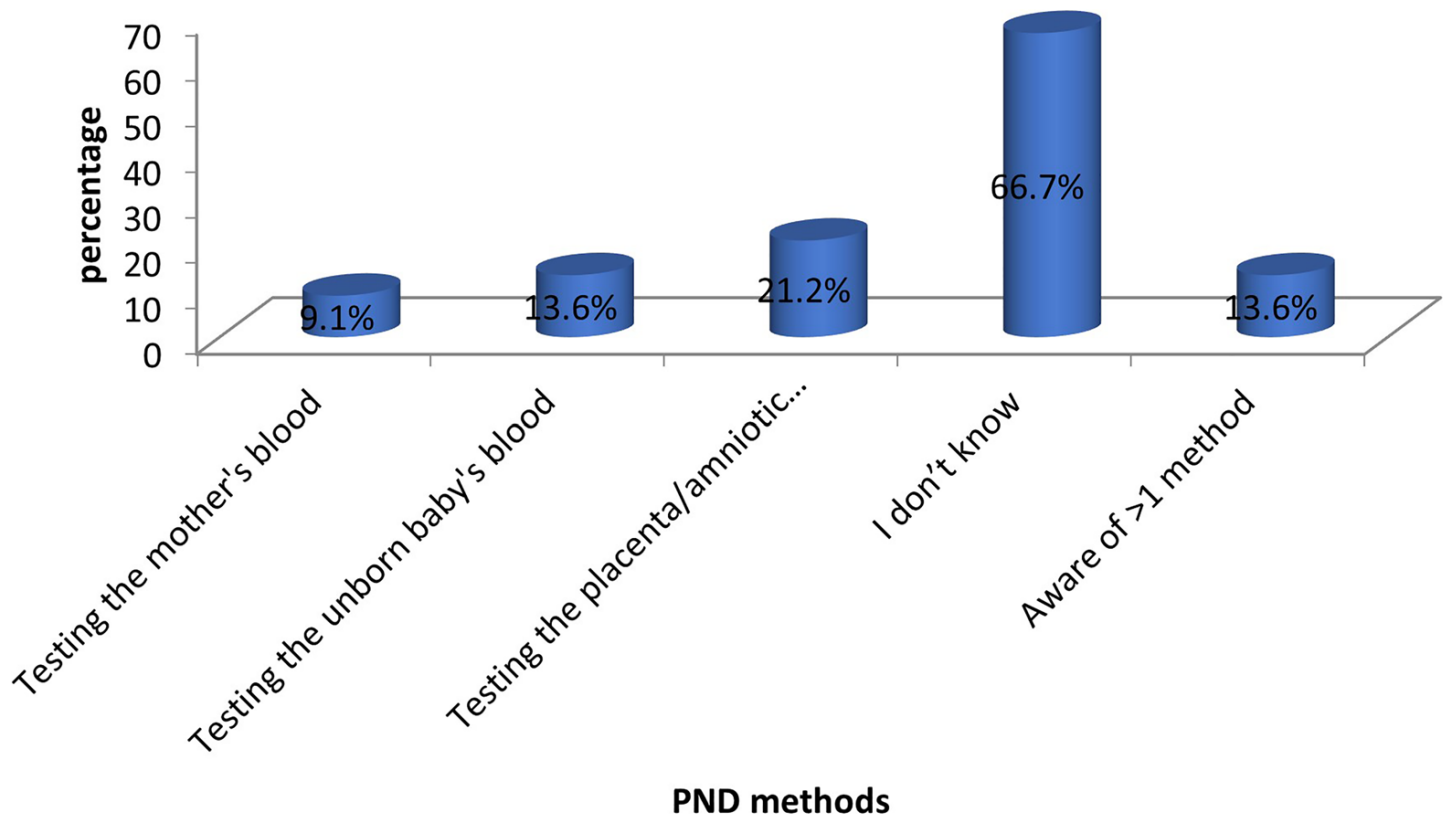
Awareness of PND methods among mothers of children with SCD. The bars represent the methods of SCD PND known to the participants.

The majority of the mothers (44 out of 66) were unaware of the methods that can be used for PND. Among those who were aware, chorionic villus sampling or amniocentesis was the most commonly known method (14 out of 66). Only 13.6% (9 out of 66) of the mothers knew more than one method of PND. These findings are depicted in Figure 2.

Only one mother (0.3%) had ever undergone a form of PND of SCD. However, when informed about PND of SCD, 93.3% of the mothers expressed willingness to accept the test. Among those who were not willing to accept PND, 45% were willing to accept the fate of having another child with SCD. These findings are detailed in Table 3.

**Table 3.** Acceptability of PND for SCD among mothers.

Variable	Frequency (%) n=297
**Ever had PND for SCD**	
Yes	1 (0.3)
No	298 (99.7)
**Willingness to accept PND in the future**	**n=297**
Yes	277 (93.3)
No	20 (6.7)
**Reasons for unwillingness to accept** **PND in the future**	**n=20**
I will accept my fate	9 (45.0)
No reason stated	11 (55.0)

Mother’s educational level, having a child experiencing at least one crisis per month, and receiving at least one blood transfusion or hospital admission in the past year were significantly associated with willingness to accept PND for SCD (p<0.05). These associations are presented in Table 4.

**Table 4.** Factors associated with the willingness to accept PND for SCD in future pregnancies.

	Frequency (%) n=297		
Factor	Will accept PND	Will not accept PND	p-value
**Educational level**			
Tertiary	57	9	**0.011**
Below tertiary	220	11	
Personal source of income			
Yes	116	9	0.785
No	161	11	
Place of residence			
Semi-urban/urban	202	16	0.489
Rural	75	4	
**No. of SCD-related death**			
≥1	57	5	0.638
0	220	15	
**Average crisis per month**			
≥1	147	4	**0.005**
0	130	16	
**SCD-related blood transfusion in** **the last one year**			
≥1	128	4	**0.034**
0	149	16	
**SCD-related hospital admission in** **the last one year**			
≥1	161	3	**0.000**
0	116	17	
**No. of children affected with SCD**			
≥2	167	9	0.158
1	110	11	

## Discussion

The global number of people with sickle cell disease (SCD) has increased by 41.4% over the past 21 years ^7^. The birth prevalence of SCD among children under one-year-old is reported to be highest in Africa, ranging from 643 to 2800 per 100,000, with particularly high mortality rates ^8^. This highlights the urgent need for effective SCD control measures in SSA to reduce associated morbidity and mortality. PND is one of the reproductive options available to couples at high risk of transmitting a serious genetic disorder. Awareness and utilization of PND have increased in developed countries, where it is routinely offered as part of informed reproductive choices, but remains low in Africa, which carries the highest burden of the disease ^9^.

This study found a low level of awareness of PND for SCD, which aligns with findings by Olutanya *et al.* in Southwestern Nigeria ^10^. Similarly, Munung *et al.* in a multi-country study of people directly affected by SCD, reported a low level of awareness of PND ^11^. This low level of awareness mirrors the general awareness of prenatal diagnosis for other genetic conditions in various study populations, such as pregnant women ^12– 14^. The widespread poor awareness of PND for genetic disorders could be attributed to a lack of facilities and expertise for PND in Nigeria and the reluctance of some healthcare workers to recommend PND for SCD. A study among physicians found that nearly two-thirds would not recommend PND for at-risk couples ^10^. This also highlights the low uptake of PND for SCD in this study, with only one mother having undergone PND in a previous pregnancy, as reported in a similar study in Nigeria ^14^. In contrast, Okechukwu *et al.* found higher awareness of PND (52.2%) among parents of SCD children in South-South Nigeria ^15^.

In this study, the internet and social media were not major sources of information about PND for SCD. In the digital era, this emphasizes the need for organisations responsible for SCD control to utilise these media to disseminate accurate and easily understandable information.

Following information on PND for SCD provided to participants in this study, the majority expressed a willingness to accept PND, consistent with reports by Ampumah *et al.* from Ghana and Wonkam *et al.* from Cameroon ^16, 17^. However, Olatunya *et al.* and Okechukwu reported lower acceptability rates in similar populations ^15^. This could be due to differences in the intrinsic characteristics of the study populations. In the study by Olatunya, 97% of participants had only one affected child, whereas more than half of the mothers in this study had more than one affected child. Additionally, mothers in this study had experienced SCD-related deaths five times more frequently than participants in Okechukwu’s study. Caring for a person with SCD carries a significant psychological, financial, and physical burden ^18^. This burden is even greater when more than one child in a family is affected by SCD, which may influence mothers' attitudes toward the acceptability of PND for SCD.

In this study, nearly half of the mothers who were unwilling to accept PND cited a willingness to accept the risk of having another affected child. Acceptance of one’s fate is closely associated with religious belief, similar to findings from southern Nigeria ^15^. Religion is a strong factor in the decision-making process for prenatal screening ^19^. This study found that severe manifestations of the disease evident by frequent crises, blood transfusions, and hospital admissions were associated with willingness to accept PND. The psychosocial burden faced by these mothers of affected children does not exist in other studied populations, such as healthcare providers or students ^20^. Cultural, religious, and personal concerns about pregnancy termination are factors associated with willingness to accept PND for SCD ^11, 19^. Ampumah *et al.* in Ghana reported that parents of children living with SCD were more likely to use PND results to prepare for the birth of the child, and deciding to accept PND did not necessarily mean using that information to terminate the pregnancy ^16^. Munung *et al.* in Ghana found a lower preference for PND as a SCD control measure largely due to its complex decision-making process and higher risk of stigmatization which are influenced by religious and cultural aspects attached to the termination of an affected pregnancy that can follow a PND ^11^. In a resource-poor country where the resources to establish widespread PND is lacking; a populace whom cultural and religious beliefs influence their decision making; a restrictive abortion law and high burden of SCD-related morbidity and mortality, there is need to strengthen other less expensive control measures in the countries like premating/premarital counseling and testing and newborn screening. In the Nigerian setting, awareness and acceptance of premarital screening for SCD are already relatively high ^21^, and studies have shown that many African individuals hold positive attitudes toward genetic counselling ^22^. Evidence from a multi-African country study by Munung *et al*. further suggests that adolescents may be an ideal target group for early sickle cell trait screening, given their openness to receiving genetic information before marriage decisions are made ^11^. This indicates a valuable opportunity to expand low-cost control measures. In contexts where religious leaders play a central role in guiding social and marital decisions, their involvement could significantly enhance the uptake and acceptability of other control measures like premarital screening. Engaging community faith structures—for example, offering voluntary carrier testing through local mosques or churches—may be culturally appropriate and effective. Religious leaders often hold strong influence and have been shown to express supportive attitudes toward premarital SCD screening, and they could play a constructive role in communicating the health implications of two carriers marrying ^23^. Cell-free fetal DNA testing in maternal serum is currently unavailable in Kaduna, and only a few centres offer the service nationwide, at prohibitively high costs.

Educational level was the only socio-demographic variable of mothers associated with willingness to accept PND, similar to findings by Mattei *et al.* in a recent systematic review ^24^. Other studies have also reported that educational level is generally associated with willingness to accept PND, not just for SCD ^25, 26^.

This study derives its strengths from the use of a large sample size; use of a relevant target population of women with a strong stake in control measures of SCD like PND; provides valuable insights into public health strategies and interventions in the control of SCD in a country with the highest burden of SCD and thus could inform future research and policy.

Limitations of this study include the use of a convenience sampling method as it does not randomly select mothers from the target population; reliance on self-reported data where the respondents may not truly reveal their beliefs and attitudes and being a single-centred study where the respondents were Muslims and of Hausa ethnicity which could limit diversity of perspectives represented in this study.

## Conclusion

Only one-fifth of at-risk mothers were aware of PND as a control measure for SCD, and actual use of PND was very low. Most mothers expressed a willingness to accept PND and religious belief was the main reason for declining PND. Mother’s educational status and the severity of the child's disease were associated with a willingness to accept PND.

## Ethics and consent

Ethical approval (ABUTHZ/HREC/H32/2022) was obtained from the Health Research Ethics Committee of ABUTH, Zaria, on 14 September 2022. Participants were provided with adequate information, and informed verbal consent was obtained from all the study participants before the questionnaires were administered. Verbal consent was used because it has been established in a national document that Kaduna state (where the study was conducted) has a low literacy level, with only 18% of the female population reported to have attained secondary or higher education (National Health Demographic Survey, 2018). The study also posed a no-minimal risk to the participants and the information collected was de-identified. The scope of the study and process involved was discussed with each participant. The participant was allowed the opportunity to ask questions and her concerns addressed if any before giving consent. The questionnaires were de-identified, and confidentiality was strictly maintained. Collected hard copy data was securely stored, and only the principal investigator had access to the input data on a computer.

## Data Availability

The data underlying this article are openly available in the Figshare repository under a CC BY 4.0. The dataset can be accessed via the following DOI 10.6084/m9.figshare.27195288 ^6^. Data are available under the terms of the CC BY 4.0

## References

[1] Regional Committee for Africa: Sickle-Cell Disease: a strategy for the WHO African region.Geneva: World Health Organization,2011. Reference Source

[2] Federal Ministry of Health: National guideline for control and management of Sickle Cell Disease.Abuja: Federal Ministry of Health,2014. Reference Source

[3] Grosse SD Odame I Atrash HK : Sickle Cell Disease in Africa: a neglected cause of early childhood mortality. *Am J Prev Med.* 2011;41(6 Suppl 4):S398–405. 10.1016/j.amepre.2011.09.013 22099364 PMC3708126

[4] Kato GJ Piel FB Reid CD : Sickle Cell Disease. *Nat Rev Dis Prim.* 2018;4: 18010. 10.1038/nrdp.2018.10 29542687

[5] Ojewunmi OO Adeyemo TA Ayinde OC : Current perspectives of Sickle Cell Disease in Nigeria: changing the narratives. *Expert Rev Hematol.* 2019;12(8):609–620. 10.1080/17474086.2019.1631155 31195888

[6] Aliyu RM Adebiyi NM Sada SI : Awareness and acceptability of prenatal diagnosis of Sickle Cell Disease among mothers of affected children in a Northern Nigerian Teaching Hospital. [Dataset], Figshare.2024. 10.6084/m9.figshare.27195288 PMC12775652 41509890

[7] GBD 2021 Sickle Cell Disease Collaborators: Global, regional, and national prevalence and mortality burden of Sickle Cell Disease, 2000-2021: a systematic analysis from the Global Burden of Disease Study 2021. *Lancet Haematol.* 2023;10(8):e585–e599. 10.1016/S2352-3026(23)00118-7 37331373 PMC10390339

[8] Colombatti R Hegemann I Medici M : Correction: Colombatti et al. Systematic Literature Review Shows Gaps in Data on Global Prevalence and Birth Prevalence of Sickle Cell Disease and Sickle Cell Trait: Call for Action to Scale Up and Harmonize Data Collection. *J. Clin. Med. 2023, 12, 5538*. *J Clin Med.* 2024;13(10):2893. 10.3390/jcm13102893 38792559 PMC11122412

[9] Nzekwue C Ogueh O : Prenatal diagnosis and preimplantation genetic diagnosis for Sickle Cell Disease in Africa. *J Glob Med.* 2022;2(1):e75. 10.51496/jogm.v2.75

[10] Olatunya OS Babatola AO Ogundare EO : Perceptions and practice of early diagnosis of Sickle Cell Disease by parents and physicians in a Southwestern state of Nigeria. *Sci World J.* 2020;2020: 4801087. 10.1155/2020/4801087 32549799 PMC7281802

[11] Munung NS Kamga KK Treadwell MJ : Perceptions and preferences for genetic testing for Sickle Cell Disease or trait: a qualitative study in Cameroon, Ghana and Tanzania. *Eur J Hum Genet.* 2024;32(10):1307–1313. 10.1038/s41431-024-01553-7 38374470 PMC11499917

[12] Ahmed Y Panti AA Umar A : Knowledge and acceptability of prenatal diagnosis among pregnant women attending antenatal clinic in a tertiary health institution in Sokoto, Nigeria. *Int J Reprod Contracept Obstet Gynecol.* 2021;10(12):3678–84. 10.18203/2320-1770.ijrcog20213830

[13] Ogamba CF Babah OA Roberts AA : Knowledge, attitudes, and decision making towards prenatal testing among antenatal clinic attendees in Lagos University Teaching Hospital: an institution-based cross-sectional study. *Pan Afr Med J.* 2021;39:106. 10.11604/pamj.2021.39.106.23667 34512842 PMC8396387

[14] Adenmosun O Andrew M Taiwo O : Knowledge and perception of pregnant women on control measures for sickle cell disorder in South-Western Nigeria. *Int J Med Sci Health Res.* 2018;2(2):200–12. Reference Source

[15] Okechukwu C : Prenatal diagnosis in Sickle Cell Disease: in the eyes of the couple at risk. *J Adv Med Med Res.* 2020;32(10):65–71. 10.9734/JAMMR/2020/v32i1030520

[16] Ampomah MO Atkin K Flemming K : The perception of parents with a child with Sickle Cell Disease in Ghana towards prenatal diagnosis. *J Community Genet.* 2022;13(6):587–95. 10.1007/s12687-022-00609-9 36197646 PMC9681951

[17] Wonkam A Njamnshi AK Mbanya D : Acceptability of prenatal diagnosis by a sample of parents of sickle cell anemia patients in cameroon (sub-Saharan Africa). *J Genet Couns.* 2011;20(5):476–85. 10.1007/s10897-011-9372-y 21604069

[18] Beli II Ali LA Onuoha CC : Socio-economic burden of Sickle Cell Disease on families attending sickle cell clinic in Kano State, Northwestern Nigeria. *Glob Pediatr Health.* 2024;9: 100193. 10.1016/j.gpeds.2024.100193

[19] Ahmed S Atkin K Hewison J : The influence of faith and religion and the role of religious and community leaders in prenatal decisions for sickle cell disorders and thalassaemia major. *Prenat Diagn.* 2006;26(9):801–9. 10.1002/pd.1507 16927359

[20] Animasahun BA Nwodo U Njokanma OF : Prenatal screening for sickle cell anemia: awareness among health professionals and medical students at the Lagos University Teaching Hospital and the concept of prevention by termination. *J Pediatr Hematol Oncol.* 2012;34(4):252–6. 10.1097/MPH.0b013e31824e3109 22538322

[21] Ugwu NI : Pre-marital screening for sickle cell haemoglobin and genetic counseling: awareness and acceptability among undergraduate students of a Nigerian university. *Int J Med Biomed Res.* 2016;5(1):43–49. 10.14194/ijmbr.5.1.6

[22] Akinola A Kaushik M Constance MS : Sickle Cell Disease knowledge and attitude towards premarital genetic counselling of African students in Africa: a systematic review. *IJRAR.* 2020;7(3).

[23] Abubakar SB Abdulqadir I Magaji BA : Knowledge, attitude and perception of traditional and religious leaders on pre-marital screening for Sickle Cell Disease in Sokoto. *Int J Med Public Health.* 2019;9(2):36–41. 10.5530/ijmedph.2019.2.10

[24] Di Mattei V Ferrari F Perego G : Decision-making factors in prenatal testing: a systematic review. *Health Psychol Open.* 2021;8(1): 2055102920987455. 10.1177/2055102920987455 33489303 PMC7809316

[25] Arumugam S Kalluri SS Sharmila V : Acceptability of prenatal screening tests among expectant mothers in India: insights and implications for public health. *Cureus.* 2024;16(5): e61246. 10.7759/cureus.61246 38939276 PMC11210580

[26] Adekanbi AOA Olayemi OO Fawole AO : The knowledge base and acceptability of prenatal diagnosis by pregnant women in Ibadan. *Afr J Reprod Health.* 2014;18(1):127–32. 24796177

