# Supplementary material for: Awareness and Acceptability of Prenatal Diagnosis of Sickle Cell Disease among Mothers of Affected Children in a Northern Nigerian Teaching Hospital
Source: Open Res Eur. 2026 Jan 22;5:278. Originally published 2025 Sep 10. [Version 3] doi: 10.12688/openreseurope.18700.3 (PMC12775652; doi:10.12688/openreseurope.18700.3)
Supplement: Peer review history

# Reviewer report: Reviewer response for version 3

**DOI:** 10.21956/openreseurope.24683.r68550

**Published:** 2026-01-24

**Authors:** Lori C Jordan

**Affiliations:** 1 Vanderbilt University Medical Center, Nashville, Tennessee, USA

I have no additional comments. Thank you.

Is the study design appropriate and does the work have academic merit?

Yes

Is the work clearly and accurately presented and does it cite the current literature?

Yes

If applicable, is the statistical analysis and its interpretation appropriate?

Yes

Are all the source data underlying the results available to ensure full reproducibility?

Yes

Are the conclusions drawn adequately supported by the results?

Yes

Are sufficient details of methods and analysis provided to allow replication by others?

Yes

Reviewer Expertise:

sickle cell disease - neurological complications

I confirm that I have read this submission and believe that I have an appropriate level of expertise to confirm that it is of an acceptable scientific standard.

# Reviewer report: Reviewer response for version 2

**DOI:** 10.21956/openreseurope.23958.r65982

**Published:** 2026-01-08

**Authors:** Miguel Brito

**Affiliations:** 1 Lisbon School of Health Technology, Lisbon, Portugal

The manuscript “Awareness and Acceptability of Prenatal Diagnosis of Sickle Cell Disease among Mothers of Affected Children in a Northern Nigerian Teaching Hospital” aims to evaluate the awareness and acceptability of PND for SCD among mothers of children with SCD. It is indeed a fascinating and important area of research. It is a well-written manuscript, well-organised, and contains important information. The sample size is acceptable, and in general it is a good methodology.

 Minor points:

 In the Introduction, the authors refer to “The non-invasive prenatal test screens for fetal SCD using cell-free fetal DNA from maternal plasma, eliminating the risk of miscarriage associated with invasive tests3,4. This method also allows for earlier screening than conventional invasive tests.” However, it should be mentioned that this is not yet a diagnostic validated method, or al least mention if it was already validated.

 There is no information if any of the mothers is an SCD patient, and if any of the mothers is pregnant, since if the authors have that information, they should mention it, and analyse if the awareness and acceptability of PND is different on that specific group.

 In sample size determination, the authors refer that in Olatunya's paper the prevalence of awareness was 0,25%, however, I believe they want o mention 25% (not 0,25%, or at least 0,25 in 1 to zero).In the manuscript, the awareness was about 22,2%. The sample size is well determined, since they used the 25% in the calculation.

 The authors mention that “ *Information on PND for SCD*” was given, and after that, the majority of the mothers accepted the PND idea. What information was given? Is there a document or a brochure shown to the patients? Was it always the same person to give the information? The authors mention that education is very low in that region, so the understanding of the new concepts could be different.

 The “Ethics and consent” paragraph is repeated in methodology and in the end of the manuscript

Is the study design appropriate and does the work have academic merit?

Yes

Is the work clearly and accurately presented and does it cite the current literature?

Yes

If applicable, is the statistical analysis and its interpretation appropriate?

Yes

Are all the source data underlying the results available to ensure full reproducibility?

Yes

Are the conclusions drawn adequately supported by the results?

Yes

Are sufficient details of methods and analysis provided to allow replication by others?

Partly

Reviewer Expertise:

SCD genetics

I confirm that I have read this submission and believe that I have an appropriate level of expertise to confirm that it is of an acceptable scientific standard.

# Response

**Published:** 2026-01-12

**Authors:** Rabi'at Muhammad Aliyu

**Affiliations:** Obstetrics and Gynaecology, Ahmadu Bello University, Zaria, Kaduna, Nigeria

Thank you for the comments.

1. The comment on cell-free DNA testing in PND has been addressed.
2. The study did not seek the pregnancy status or genotype of the mothers during data collection.
3. The awareness level quoted from Olatunya’s study has been corrected to 25%.
4. The questionnaires were interviewer-administered by two trained research assistants. Any participant who was not aware of PND for SCD was verbally provided with information on the existence of the procedure and the various methods that can be employed.

# Reviewer report: Reviewer response for version 2

**DOI:** 10.21956/openreseurope.23958.r65981

**Published:** 2026-01-07

**Authors:** Ravindra Kumar

**Affiliations:** 1 ICMR-National Institute of Research in Tribal Health, Jabalpur, India

The manuscript is well written. However, certain corrections are needed. 

 In table 1, the percentage in Islam is 97.0 not 97,0

 In table 2, hemglobin genotype may be replaced as genotype. The method used to assess the genotype is not mentioned in the manuscript. 

 In Table 4, the percentage is not mentioned. 

 In discussion, how this information will help in policy making and increase in PND services should be discussed 

Is the study design appropriate and does the work have academic merit?

Partly

Is the work clearly and accurately presented and does it cite the current literature?

Yes

If applicable, is the statistical analysis and its interpretation appropriate?

Partly

Are all the source data underlying the results available to ensure full reproducibility?

Yes

Are the conclusions drawn adequately supported by the results?

Yes

Are sufficient details of methods and analysis provided to allow replication by others?

Yes

Reviewer Expertise:

Hemoglobinopathies, Public Health

I confirm that I have read this submission and believe that I have an appropriate level of expertise to confirm that it is of an acceptable scientific standard, however I have significant reservations, as outlined above.

# Response

**Published:** 2026-01-12

**Authors:** Rabi'at Muhammad Aliyu

**Affiliations:** Obstetrics and Gynaecology, Ahmadu Bello University, Zaria, Kaduna, Nigeria

Thank you for the comments.

1. In Table 1, a correction to 97.0 was effected.
2. In Table 2, haemoglobin has been removed.
3. The method of assessing genotype is by alkaline electrophoresis and has been included in the study setting of methods
4. The percentages have been added in Table 4
5. Discussion about study findings and policy-making has been enriched

# Reviewer report: Reviewer response for version 2

**DOI:** 10.21956/openreseurope.23958.r65788

**Published:** 2026-01-07

**Authors:** Lori C Jordan

**Affiliations:** 1 Vanderbilt University Medical Center, Nashville, Tennessee, USA

This is an excellent paper, and I appreciated the author’s clarifications. I recommend adding the information below to the manuscript rather than just responding to my comment

 The authors responded:

 Tertiary education in this study referred to an educational level higher than secondary school

 But, they did not add this to the manuscript. Many readers will not understand this terminology. It’s not standard country to country.  Recommend adding either as a foot note to the table or a sentence in the results.

 For example, the first paragraph of the results says: Further details are provided in Table 1. Could Add after this sentence: Tertiary education refers to an educational level higher than secondary school and was achieved by 22.2% of mothers participating in this study.

Is the study design appropriate and does the work have academic merit?

Yes

Is the work clearly and accurately presented and does it cite the current literature?

Yes

If applicable, is the statistical analysis and its interpretation appropriate?

Yes

Are all the source data underlying the results available to ensure full reproducibility?

Yes

Are the conclusions drawn adequately supported by the results?

Yes

Are sufficient details of methods and analysis provided to allow replication by others?

Yes

Reviewer Expertise:

sickle cell disease - neurological complications

I confirm that I have read this submission and believe that I have an appropriate level of expertise to confirm that it is of an acceptable scientific standard.

# Reviewer report: Reviewer response for version 2

**DOI:** 10.21956/openreseurope.23958.r65979

**Published:** 2026-01-06

**Authors:** Aisha Galandanci

**Affiliations:** 1 Hematology, Aminu Kano Teaching Hospital (Ringgold ID: 376586), Kano, Kano, Nigeria; 2 Global Pediatric Medicine, St Jude Children's Research Hospital, Memphis, TN, USA

This study is particularly significant, especially as Nigeria bears the highest global burden of SCD. While the findings provide valuable insights into barriers to PND uptake, the absence of a discussion on the cost of PND relative to the mother's income is a notable limitation. In low-resource settings, out-of-pocket costs are often a major factor influencing healthcare decisions. It would be useful to explore whether lack of affordability or cost awareness may have contributed to the low acceptability rates observed.

 The study could also benefit from addressing the limited autonomy many women experience in reproductive decision-making, particularly those with less formal education. In many Nigerian households, such decisions are made by husbands or extended family members, which may explain the reluctance to accept PND even when it is available.

 In the discussion, the authors rightly note that in a resource-poor country where widespread access to PND is limited, and where cultural, religious, and legal factors influence decision-making, there is an urgent need to strengthen other cost-effective control measures such as premarital counseling, carrier testing, and newborn screening. Building on this, the manuscript could consider emphasizing collaboration with religious leaders to provide culturally sensitive education on SCD and Islamic perspectives on PND. Religious institutions such as mosques could play an important role in increasing awareness and trust. Similarly, expanding SCD education and screening awareness to universities and public institutions may help reach younger individuals before marriage or childbearing.

 Finally, minor editorial revisions are recommended to improve clarity  including removing redundant content (e.g., the repeated ethics section) and ensuring consistency in abbreviation usage (e.g., defining SCD once). These adjustments would enhance the manuscript’s overall impact and readability.

Is the study design appropriate and does the work have academic merit?

Yes

Is the work clearly and accurately presented and does it cite the current literature?

Yes

If applicable, is the statistical analysis and its interpretation appropriate?

Yes

Are all the source data underlying the results available to ensure full reproducibility?

Yes

Are the conclusions drawn adequately supported by the results?

Partly

Are sufficient details of methods and analysis provided to allow replication by others?

Yes

Reviewer Expertise:

1)Hemoglobinopathies, particularly Sickle Cell Disease (SCD), Epidemiology and disease burden, Newborn screening (NBS) for SCD, prevention strategies and genetic counseling, community health education and awareness,2) Quantitative and qualitative research methods, caregiver experiences and health-seeking behavior, sociocultural and religious influences on health decisions,

I confirm that I have read this submission and believe that I have an appropriate level of expertise to confirm that it is of an acceptable scientific standard.

# Response

**Published:** 2026-01-12

**Authors:** Rabi'at Muhammad Aliyu

**Affiliations:** Obstetrics and Gynaecology, Ahmadu Bello University, Zaria, Kaduna, Nigeria

Thank you for the insightful comments.

1.  The study found a high rate of acceptability (93.3%) after receiving information about prenatal diagnosis. Nearly half (45%) of the women who were not willing to accept prenatal diagnosis cited religious beliefs as the reason, rather than the cost
2. The role of religious leaders in offering premarital counselling has been expanded and referenced.
3. The role of health educating younger individuals in the control of SCD has been addressed.
4. The minor editorial revisions have been done.  

# Reviewer report: Reviewer response for version 1

**DOI:** 10.21956/openreseurope.20230.r63815

**Published:** 2025-11-25

**Authors:** Vincenzo Voi

**Affiliations:** 1 University of Turin, Turin, Italy

Peer Review Report – Open Research Europe

 Title

 Awareness and Acceptability of Prenatal Diagnosis of Sickle Cell Disease among Mothers of Affected Children in a Northern Nigerian Teaching Hospital

 General Assessment

 The manuscript addresses a highly relevant topic in the field of sickle cell disease (SCD) prevention. The study is clearly structured and uses a sample directly relevant to the research question.

 Some methodological and interpretative aspects require clarification:

 Methods – Points Requiring Revision

**Referenced text: “After obtaining informed verbal consent, every eligible participant completed a structured interviewer-administered questionnaire…”**

 Suggested revision: I agree with my previous colleague. The manuscript should report the number of mothers who declined participation. If none declined, authors should explicitly state this. Moreover, clarification is needed on the total number of children with SCD routinely followed at the clinic to contextualize the recruitment proportion.

 Results – Clarifications Needed

**Referenced text: “About one-third of the mothers were grandmultiparous...”**

 Suggested revision: Define clearly what threshold the authors used for ‘grand multiparous’. Literature definitions vary (≥5 deliveries is common).

 Define the operational threshold used in this study to classify grand multiparity. In the narrative text, however, the authors describe the mothers as “about one-third grand multiparous” with a “median parity of 3,” which seems inconsistent with the ≥5 threshold reported in the table.

 If the intended definition is ≥5 deliveries, adjust the text to align with the tables. If the authors used a different definition, modify the table labels accordingly.

**Referenced text: “Below tertiary vs tertiary”**

 Suggested revision: Specify what is included under ‘tertiary education’. Indicate whether it reflects post-secondary, university-level, or vocational training.

 Discussion – Conceptual Expansion Needed

**Referenced text: “There is need to strengthen other less expensive control measures…”**

 Suggested revision: Expand this point. In regions where pregnancy termination is culturally or religiously restricted, premarital carrier screening and community-based education programs could play a critical role. Consider discussing the feasibility of partnerships with local religious and community leaders, who are often central figures in decision‑making. Examples exist in other communities (e.g., faith-based premarital genetic counseling programs).

 Discussion – Technical Clarification

**Referenced text: discussion of non-invasive prenatal diagnosis.**

 Suggested revision: The authors should clarify whether cell‑free fetal DNA testing for SCD is available in Kaduna or elsewhere in Nigeria, and whether cost or infrastructure barriers limit its use. This would give readers a realistic view of PND implementation challenges.

 Minor Editorial Suggestions

 Ensure consistency in numeration: the text reports 0.3% for undergoing PND, but 1/297 corresponds to ~0.34%—the number is correct but could be rounded more transparently.

 Consider adding a short sentence on how missing data, if any, were handled.

Is the study design appropriate and does the work have academic merit?

Yes

Is the work clearly and accurately presented and does it cite the current literature?

Yes

If applicable, is the statistical analysis and its interpretation appropriate?

Yes

Are all the source data underlying the results available to ensure full reproducibility?

Yes

Are the conclusions drawn adequately supported by the results?

Yes

Are sufficient details of methods and analysis provided to allow replication by others?

Yes

Reviewer Expertise:

Sickle Cell Disease expert.

I confirm that I have read this submission and believe that I have an appropriate level of expertise to confirm that it is of an acceptable scientific standard.

# Response

**Published:** 2025-12-05

**Authors:** Rabi'at Muhammad Aliyu

**Affiliations:** Obstetrics and Gynaecology, Ahmadu Bello University, Zaria, Kaduna, Nigeria

Thank you for your insightful comments. METHODS None of the eligible mothers declined participation. The clinic attends to an average of 50 children with SCD per week RESULT

1. Grandmultiparous women in this study referred to women who have had five or more deliveries, and the term is now used consistently.
2. Tertiary education in this study refers to additional education beyond secondary school and includes diplomas, degrees, postgraduate

DISCUSSION

1. Point on religious leaders involvement have been expanded.
2. Clarification on cell free fetal DNA has been provided

# Reviewer report: Reviewer response for version 1

**DOI:** 10.21956/openreseurope.20230.r60590

**Published:** 2025-10-08

**Authors:** Lori C Jordan

**Affiliations:** 1 Vanderbilt University Medical Center, Nashville, Tennessee, USA

This seems like an important topic and excellent subject for investigation.

 I have a few questions organized by section:

 Methods:

 The authors state: “After obtaining informed verbal consent, every eligible participant completed a structured interviewer-administered questionnaire to gather data on socio-demographic characteristics, awareness, attitude, and acceptability of prenatal diagnosis

 for SCD. The data collected was pseudo-anonymized. Recruitment took place from March to August 2023.”

 1.How many mothers declined to participate?  If none, would reword the sentence to read: every eligible participant completed a structured interviewer-administered

 Questionnaire, after obtaining informed verbal consent,  to gather data on socio-demographic characteristics…

 2.How many children with SCD are followed in the Kaduna peds SCD clinics?  One might think at least half of these children/families would have been approached within 6 months.

 Results

 Please define grand multip – from the manuscript I thought 3 pregnancies but from the table I thought it might be 5 pregnancies.

 Please define “tertiary” education.  Do the authors mean beyond high school?  Some college

 Discussion

 The authors mention “there is need to strengthen other less expensive control measures in the countries like premating/premarital counseling and testing and newborn screening”  I agree!  Could the authors say a bit more? Would it be acceptable to screen teens for SCD carrier status through a local mosque? Perhaps an Imam might communicate that it is best for 2 SCD carriers not to marry?  Just thinking that if pregnancy termination isn’t acceptable – testing prior to marriage might be best. (I believe this has been done in Jewish populations through synagogues for different severe conditions like Tay Sachs.)

 Is cell-free DNA testing available for diagnosis in Kaduna?  Is this available at many/most facilities in Nigeria? Is testing affordable?

Is the study design appropriate and does the work have academic merit?

Yes

Is the work clearly and accurately presented and does it cite the current literature?

Yes

If applicable, is the statistical analysis and its interpretation appropriate?

Yes

Are all the source data underlying the results available to ensure full reproducibility?

Yes

Are the conclusions drawn adequately supported by the results?

Yes

Are sufficient details of methods and analysis provided to allow replication by others?

Yes

Reviewer Expertise:

sickle cell disease - neurological complications

I confirm that I have read this submission and believe that I have an appropriate level of expertise to confirm that it is of an acceptable scientific standard, however I have significant reservations, as outlined above.

# Response

**Published:** 2025-12-05

**Authors:** Rabi'at Muhammad Aliyu

**Affiliations:** Obstetrics and Gynaecology, Ahmadu Bello University, Zaria, Kaduna, Nigeria

Thank you for your insightful comments. METHODS

1. It has been reworded. None of the eligible women declined participation in the study.
2. The Paediatric Haematology Clinic attends to an average of 50 children with sickle cell disease per week.

RESULTS

1. A grandmultipara referred in this study is a woman who has had five or more pregnancies that have been carried to at least 28 weeks of gestation.
2. Tertiary education in this study referred to an educational level higher than secondary school

DISCUSSION

1. The roles of premarital screening and religious leaders' involvement as control measures have been elaborated. In the Nigerian setting, awareness and acceptance of premarital screening for SCD are already relatively high, ^21^ and studies have shown that many African individuals hold positive attitudes toward genetic counselling. ^22^ Evidence from a multi-African country study by Munung et al. further suggests that adolescents may be an ideal target group for early sickle cell trait screening, given their openness to receiving genetic information before marriage decisions are made. ^11^ This indicates a valuable opportunity to expand low-cost control measures. In contexts where religious leaders play a central role in guiding social and marital decisions, their involvement could significantly enhance the uptake and acceptability of other control measures like premarital screening. Engaging community faith structures—for example, offering voluntary carrier testing through local mosques or churches—may be culturally appropriate and effective. Religious leaders often hold strong influence and have been shown to express supportive attitudes toward premarital SCD screening, and they could play a constructive role in communicating the health implications of two carriers marrying. ^23^
2. Cell-free fetal DNA testing in maternal serum is currently unavailable in Kaduna, and only a few centres offer the service nationwide, at prohibitively high costs.
